# Supplementary material for: Transcriptomal Insights of Heart Failure from Normality to Recovery
Source: Biomolecules. 2022 May 23;12(5):731. doi: 10.3390/biom12050731 (PMC9138767; doi:10.3390/biom12050731)
Supplement: Supplementary file 1 [file biomolecules-12-00731-s001.zip › Supplementary Figure S2 RNA quality and quantity.pdf]

Control N1

RIN= 9.3

8521904000061 [1:9]

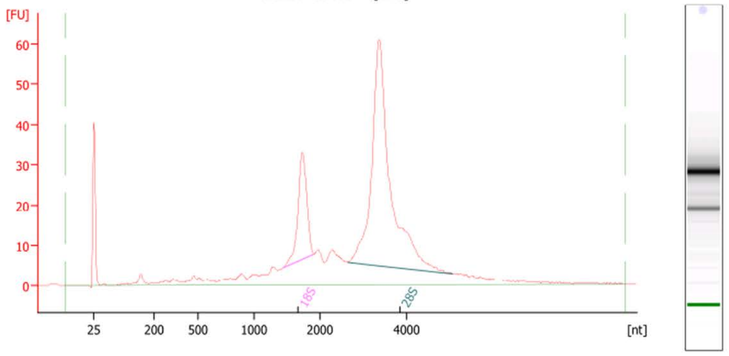

Overall Results for sample 9 : 8521904000061

RNA Area:	541.9	RNA Integrity Number (RIN):	9.3 (B.02.07)
RNA Concentration:	1,349 pg/µl	Result Flagging Color:	
rRNA Ratio [28s / 18s]:	3.8	Result Flagging Label:	RIN: 9.30

Fragment table for sample 9 : 8521904000061

Name	Start Size [nt]	End Size [nt]	Area	% of total Area
18S	1,428	1,905	58.3	10.8
28S	2,649	5,072	222.4	41.0

Control N3

RIN= 8.3

8521904000063 [1:0]

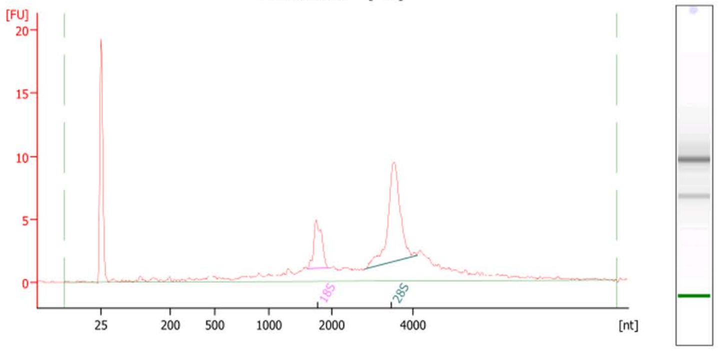

Overall Results for sample 7 : 8521904000063

RNA Area:	89.5	RNA Integrity Number (RIN):	8.3 (B.02.07)
RNA Concentration:	37 ng/µl	Result Flagging Color:	
rRNA Ratio [28s / 18s]:	2.9	Result Flagging Label:	RIN: 8.30

Fragment table for sample 7 : 8521904000063

Name	Start Size [nt]	End Size [nt]	Area	% of total Area
18S	1,622	1,962	7.2	8.1
28S	2,864	4,111	20.8	23.3

8521904000064 [1:0]

Control N4

RIN= 8.4

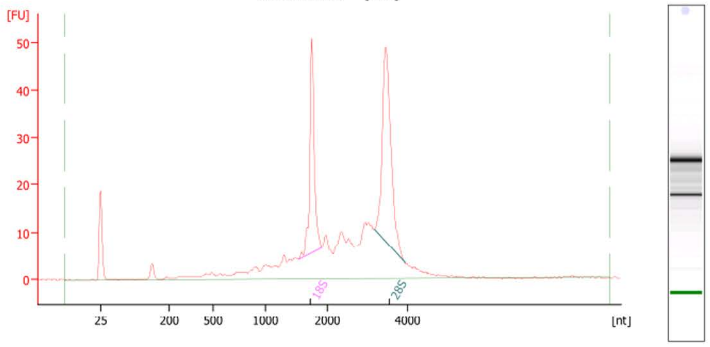

Overall Results for sample 8 : 8521904000064

RNA Area:	351.4	RNA Integrity Number (RIN):	8.4 (B.02.07)
RNA Concentration:	147 ng/µl	Result Flagging Color:	
rRNA Ratio [28s / 18s]:	1.6	Result Flagging Label:	RIN: 8.40

Fragment table for sample 8 : 8521904000064

Name	Start Size [nt]	End Size [nt]	Area	% of total Area
18S	1,540	1,916	48.6	13.8
28S	3,190	3,951	79.3	22.6

Hypertrophy H5
RIN= 8.9

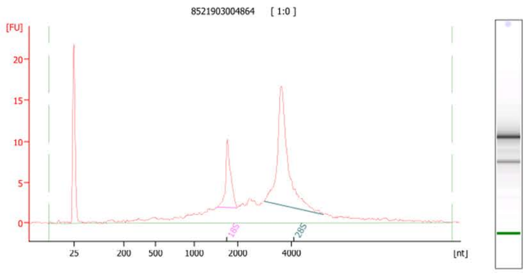

Overall Results for sample 3 : 8521903004864

RNA Area:	155.1	RNA Integrity Number (RIN):	8.9 (B.02.07)
RNA Concentration:	84 ng/µl	Result Flagging Color:	
rRNA Ratio [28s / 18s]:	3.1	Result Flagging Label:	RIN: 8.90

Fragment table for sample 3 : 8521903004864

Name	Start Size [nt]	End Size [nt]	Area	% of total Area
18S	1,527	1,985	14.6	9.4
28S	3,012	5,175	45.3	29.2

Hypertrophy H6
RIN= 8.8

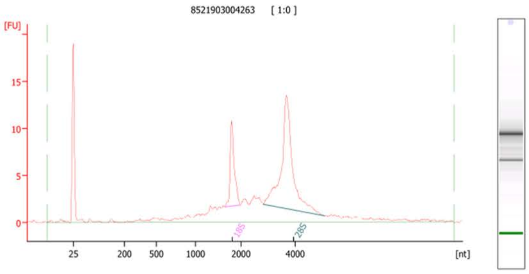

Overall Results for sample 2 : 8521903004263

RNA Area:	126.7	RNA Integrity Number (RIN):	8.8 (B.02.07)
RNA Concentration:	51 ng/µl	Result Flagging Color:	
rRNA Ratio [28s / 18s]:	3.3	Result Flagging Label:	RIN: 8.80

Fragment table for sample 2 : 8521903004263

Name	Start Size [nt]	End Size [nt]	Area	% of total Area
18S	1,639	1,985	11.8	9.3
28S	2,840	5,061	38.6	30.5

Hypertrophy H7
RIN= 9.8

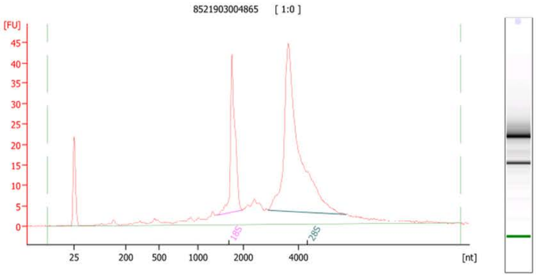

Overall Results for sample 4 : 8521903004865

RNA Area:	396.5	RNA Integrity Number (RIN):	9.8 (B.02.07)
RNA Concentration:	214 ng/µl	Result Flagging Color:	
rRNA Ratio [28s / 18s]:	2.9	Result Flagging Label:	RIN: 9.80

Fragment table for sample 4 : 8521903004865

Name	Start Size [nt]	End Size [nt]	Area	% of total Area
18S	1,412	1,969	57.9	14.6
28S	2,921	5,696	168.4	42.5

Hypertrophy S1H
RIN= 8.5

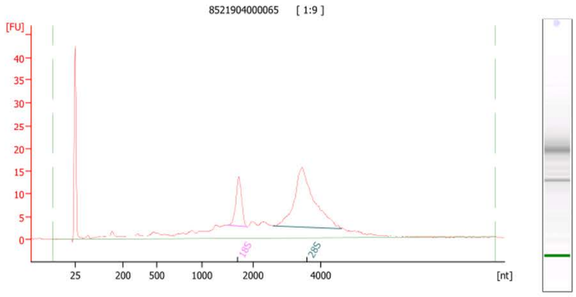

Overall Results for sample 11 : 8521904000065

RNA Area:	239.2	RNA Integrity Number (RIN):	8.5 (B.02.07)
RNA Concentration:	595 pg/µl	Result Flagging Color:	
rRNA Ratio [28s / 18s]:	3.5	Result Flagging Label:	RIN: 8.50

Fragment table for sample 11 : 8521904000065

Name	Start Size [nt]	End Size [nt]	Area	% of total Area
18S	1,504	1,878	19.6	8.2
28S	2,584	4,584	68.9	28.8

Dilated D11
RIN= 8.4

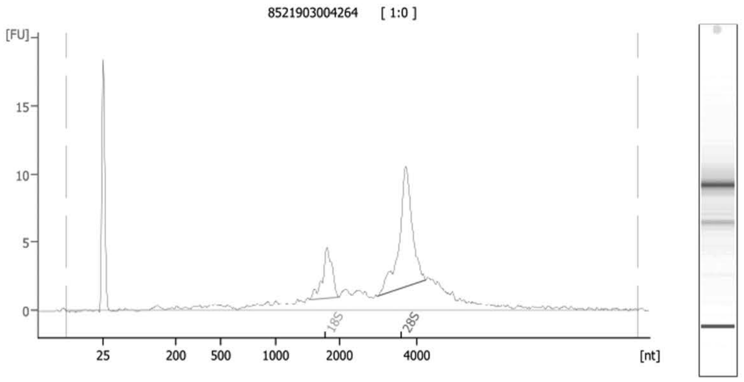

Overall Results for sample 3 : 8521903004264

RNA Area:	90.1	RNA Integrity Number (RIN):	8.4 (B.02.07)
RNA Concentration:	36 ng/µl	Result Flagging Color:	
rRNA Ratio [28s / 18s]:	2.9	Result Flagging Label:	RIN: 8.40

Fragment table for sample 3 : 8521903004264

Name	Start Size [nt]	End Size [nt]	Area	% of total Area
18S	1,532	1,995	8.1	9.0
28S	3,018	4,238	23.8	26.4

Dilated D12
RIN= 6.2

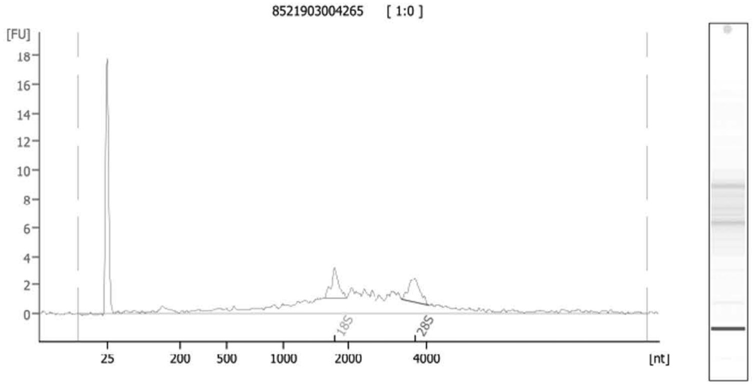

Overall Results for sample 4 : 8521903004265

RNA Area:	54.0	RNA Integrity Number (RIN):	6.2 (B.02.07)
RNA Concentration:	22 ng/µl	Result Flagging Color:	
rRNA Ratio [28s / 18s]:	1.0	Result Flagging Label:	RIN: 6.20

Fragment table for sample 4 : 8521903004265

Name	Start Size [nt]	End Size [nt]	Area	% of total Area
18S	1,619	1,980	3.7	6.9
28S	3,371	4,065	3.8	7.0

Dilated S9D
RIN= 9.4

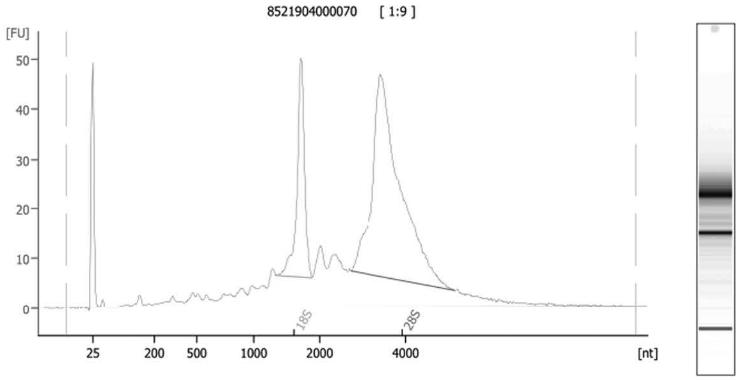

Overall Results for sample 5 : 8521904000070

RNA Area:	655.3	RNA Integrity Number (RIN):	9.4 (B.02.07)
RNA Concentration:	1,378 pg/µl	Result Flagging Color:	
rRNA Ratio [28s / 18s]:	2.9	Result Flagging Label:	RIN: 9.40

Fragment table for sample 5 : 8521904000070

Name	Start Size [nt]	End Size [nt]	Area	% of total Area
18S	1,345	1,889	83.5	12.7
28S	2,731	5,135	241.9	36.9

Dilated D13
RIN=9.8

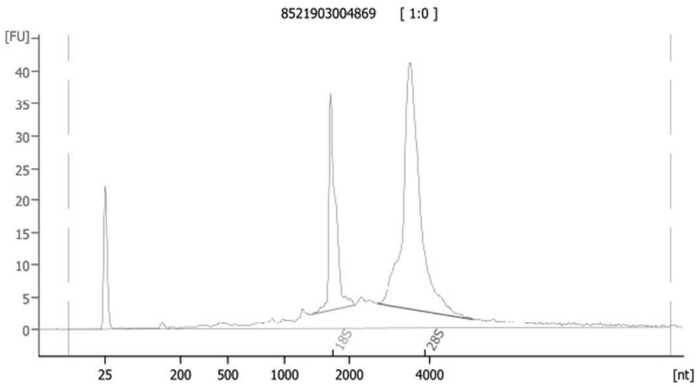

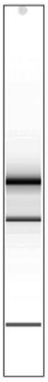

Overall Results for sample 8 : 8521903004869

RNA Area:	314.6	RNA Integrity Number (RIN):	9.8 (B.02.07)
RNA Concentration:	170 ng/µl	Result Flagging Color:	
rRNA Ratio [28s / 18s]:	2.5	Result Flagging Label:	RIN: 9.80

Fragment table for sample 8 : 8521903004869

Name	Start Size [nt]	End Size [nt]	Area	% of total Area
18S	1,403	2,140	51.9	16.5
28S	2,697	5,105	130.5	41.5

LV failure F14
RIN= 8.1

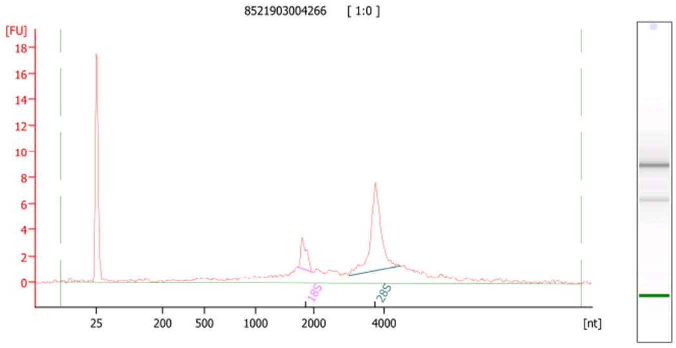

Overall Results for sample 5 : 8521903004266

RNA Area:	63.5	RNA Integrity Number (RIN):	8.1 (B.02.07, Anomaly Threshold(s) manually adapted)
RNA Concentration:	26 ng/µl		
rRNA Ratio [28s / 18s]:	4.2	Result Flagging Color:	
		Result Flagging Label:	RIN: 8.10

Fragment table for sample 5 : 8521903004266

Name	Start Size [nt]	End Size [nt]	Area	% of total Area
18S	1,729	1,981	3.7	5.8
28S	3,015	4,445	15.4	24.3

LV failure F15
RIN= 9.5

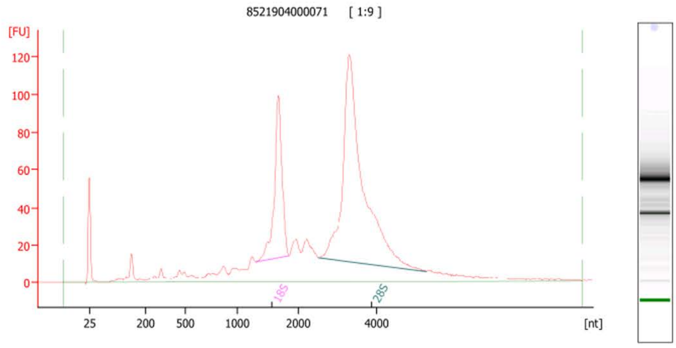

Overall Results for sample 6 : 8521904000071

RNA Area:	1,352.8	RNA Integrity Number (RIN):	9.5 (B.02.07)
RNA Concentration:	2,844 pg/µl	Result Flagging Color:	
rRNA Ratio [28s / 18s]:	2.9	Result Flagging Label:	RIN: 9.50

Fragment table for sample 6 : 8521904000071

Name	Start Size [nt]	End Size [nt]	Area	% of total Area
18S	1,302	1,850	177.9	13.1
28S	2,520	5,230	508.0	37.6

LV failure F16
RIN= 8.3

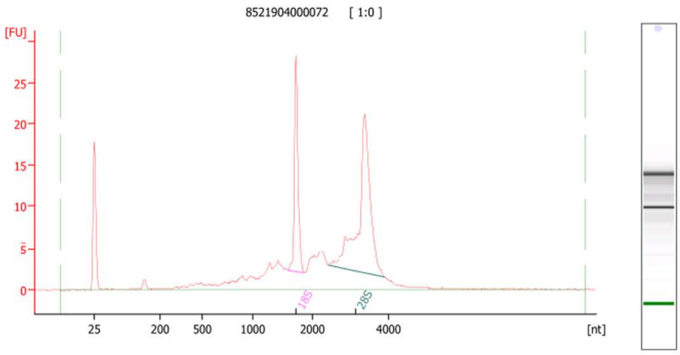

Overall Results for sample 10 : 8521904000072

RNA Area:	173.4	RNA Integrity Number (RIN):	8.3 (B.02.07)
RNA Concentration:	73 ng/µl	Result Flagging Color:	
rRNA Ratio [28s / 18s]:	2.1	Result Flagging Label:	RIN: 8.30

Fragment table for sample 10 : 8521904000072

Name	Start Size [nt]	End Size [nt]	Area	% of total Area
18S	1,592	1,870	24.1	13.9
28S	2,429	3,872	49.4	28.5

Recovery R8
RIN=8.6

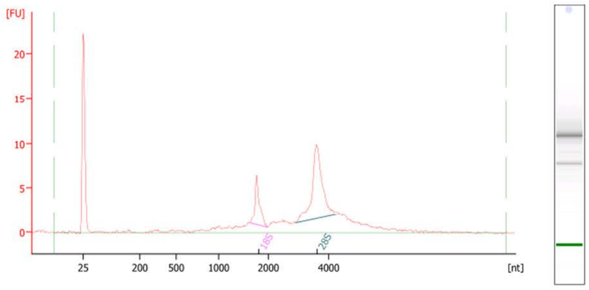

Overall Results for sample 5 : **8521903004866**

RNA Area:	81.8	RNA Integrity Number (RIN):	8.6 (B.02.07)
RNA Concentration:	44 ng/µl	Result Flagging Color:	
rRNA Ratio [28s / 18s]:	2.9	Result Flagging Label:	RIN: 8.60

Fragment table for sample 5 : **8521903004866**

Name	Start Size [nt]	End Size [nt]	Area	% of total Area
18S	1,617	1,988	7.5	9.2
28S	2,936	4,353	21.9	26.7

Recovery R9
RIN=8.4

8521903004867 [1:0]

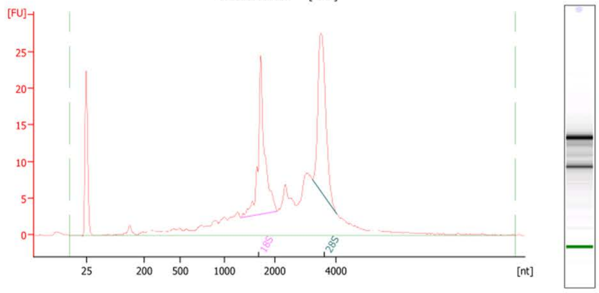

Overall Results for sample 6 : **8521903004867**

RNA Area:	246.1	RNA Integrity Number (RIN):	8.4 (B.02.07)
RNA Concentration:	133 ng/µl	Result Flagging Color:	
rRNA Ratio [28s / 18s]:	1.1	Result Flagging Label:	RIN: 8.40

Fragment table for sample 6 : **8521903004867**

Name	Start Size [nt]	End Size [nt]	Area	% of total Area
18S	1,322	2,087	41.4	16.8
28S	3,233	4,007	43.7	17.7

Recovery R10
RIN=8.4

8521903004868 [1:0]

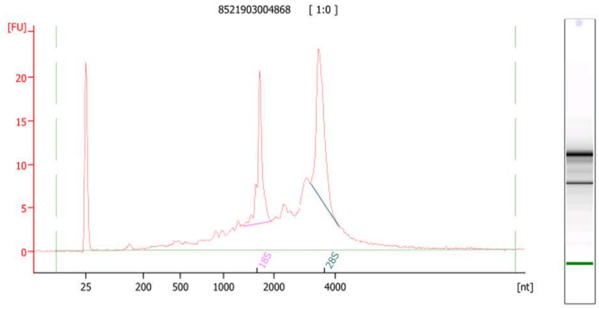

Overall Results for sample 7 : **8521903004868**

RNA Area:	218.4	RNA Integrity Number (RIN):	7.8 (B.02.07)
RNA Concentration:	118 ng/µl	Result Flagging Color:	
rRNA Ratio [28s / 18s]:	1.4	Result Flagging Label:	RIN: 7.80

Fragment table for sample 7 : **8521903004868**

Name	Start Size [nt]	End Size [nt]	Area	% of total Area
18S	1,394	1,932	24.1	11.0
28S	3,175	4,114	34.7	15.9
