# Supplementary material for: Transcriptomal Insights of Heart Failure from Normality to Recovery
Source: Biomolecules. 2022 May 23;12(5):731. doi: 10.3390/biom12050731 (PMC9138767; doi:10.3390/biom12050731)
Supplement: Supplementary file 1 [file biomolecules-12-00731-s001.zip › Supplementary Tables.pdf]

S. Table 1: List of significant genes

log2FC **(Hyp/Con)**	Symbol	log2FC **(Dil/Con)**	Symbol	log2FC **(Fail/Con)**	Symbol	log2FC **(Rec/Con)**	Symbol
5.67	TPT1	5.56	TPT1	3.82	TPT1	2.82	TARBP1
4.47	NR4A3	2.99	LOC114108841	3.79	SLN	-1.23	LOC101122330
3.87	NR4A1	2.48	LOC114108777	3.75	CD44	-2.85	MPC1
3.86	FOS	2.37	LOC101104705	3.39	TNNT1	-3.92	GON7
3.67	ATF3	2.25	FABP4	3.33	LGALS3	-4.11	LOC105605968
3.50	NPPB	2.21	SLN	2.92	TIMP1		
3.38	OTUD1	2.07	LGALS3	2.91	FABP4		
2.98	LOC114113596	2.05	TMEM205	2.91	ANKRD2		
2.98	LGALS3	2.01	TNNT1	2.79	HSPA1A		
2.91	KLF4	1.99	ADIRF	2.65	SERPINE2		
2.90	SOX17	1.91	LOC114108675	2.52	TUBB6		
2.75	RASD1	1.84	C6H4orf48	2.33	SLC39A8		
2.74	APOLD1	1.84	HSF4	2.33	UCP2		
2.67	LOC101105609	1.75	NGRN	2.28	THBS1		
2.63	ABRA	1.72	IGFBP5	2.27	ENAH		
2.62	CSRNP1	1.71	PROSER1	2.22	STK38L		
2.54	IER2	1.68	ERICH2	2.16	IGFBP4		
2.48	ID2	1.66	NOL3	2.07	CNN1		
2.45	KLF10	1.61	ID3	2.06	S100B		
2.44	DUSP1	1.57	LOC114112700	2.03	HIF1A		
2.43	MYOT	1.54	C15H11orf96	1.98	SYNPO2		
2.34	KLF2	1.52	ID1	1.98	ERRFI1		
2.31	IGFBP5	1.51	LOC114108775	1.98	ACOT9		
2.28	C15H11orf96	1.49	APOE	1.97	ATF3		
2.26	BHLHE40	1.47	GGT5	1.95	SLC1A5		
2.22	ID3	1.42	LOC114112251	1.94	LDHA		
2.20	BTG2	1.39	SRXN1	1.92	MMP2		
2.17	SNAI2	1.37	VWF	1.84	LOC114108675		
2.17	ZFP36	1.35	ID2	1.83	STK26		
2.11	SGK1	1.33	CINP	1.80	ARID5A		
2.09	OVAR-DRB1	1.33	S100B	1.79	ADAMTS1		
2.09	ARID5A	1.30	NQO2	1.77	TUBA1A		
2.01	ID1	1.28	THEM4	1.73	FKBP10		
2.00	LOC101109747	1.25	LOC114110633	1.68	PDLIM3		
1.99	MYC	1.25	JAM2	1.68	SMAD1		
1.98	SAT1	1.23	ZFP36L1	1.64	ITGAV		
1.98	CIART	1.21	APLP1	1.63	OLFML3		

1.95	CNTFR	1.17	KLF2	1.61	LRP1
1.90	C1R	1.17	CCT6A	1.59	ABRA
1.88	NPPA	1.15	KLF4	1.57	LITAF
1.87	GADD45B	1.14	FAM110D	1.56	LOC101105064
1.87	NRARP	1.08	MAP1A	1.56	MMRN1
1.84	NFIL3	1.06	PMP22	1.55	TNFRSF1B
1.83	ITPRIP	1.02	RPS3	1.53	UBQLN1
1.82	LOC101104705	1.00	TAF7	1.53	SORT1
1.82	TUBB6	-1.00	TUBA8	1.52	SKP1-2
1.81	RCAN1	-1.00	CCDC69	1.50	VWF
1.80	LOC101111832	-1.00	TUBA4A	1.48	RASSF8
1.79	MAFF	-1.03	C5H5orf63	1.48	ENPP1
1.78	SIK1	-1.04	LOC114109394	1.48	ACTR3B
1.75	LOC114108777	-1.05	COL1A1	1.45	TIMP3
1.75	LOC114108841	-1.05	DDIT4	1.45	TMEM205
1.75	LOC114118746	-1.06	TXNIP	1.43	EIF4A1
1.72	SMAD7	-1.08	LOC101114319	1.43	BAG3
1.71	KCNJ3	-1.08	HACD1	1.43	SNAI2
1.70	JUNB	-1.11	PPP1R15A	1.43	PON2
1.67	MUSTN1	-1.12	NFKBIA	1.43	ACTN1
1.67	DDIT4	-1.14	CDKN1A	1.43	LOC105608622
1.67	DZANK1	-1.14	DNAJB5	1.41	SRGN
1.66	FBXL17	-1.18	DUSP8	1.41	STAT3
1.65	NQO1	-1.19	COL3A1	1.40	ZFP36L1
1.64	HAND1	-1.20	LOC101108171	1.40	SLC9A6
1.63	NARS	-1.30	LOC101110855	1.40	SRXN1
1.62	SOCS1	-1.31	LOC101119130	1.40	SULF2
1.60	CCN1	-1.32	LOC101117554	1.39	DZANK1
1.59	IRS2	-1.33	GPT	1.37	CYLD
1.54	GEM	-1.39	GADD45G	1.36	ARF4
1.52	OVAR	-1.53	PDIA2	1.34	LOC100101238
1.52	IFRD1	-1.53	LOC101103401	1.34	SPSB1
1.50	PLVAP	-1.55	CISH	1.34	ACSL4
1.48	LOC101112038	-1.61	SUCLA2	1.34	STK17A
1.46	ARID5B	-1.65	ARHGDIG	1.33	FYTTD1
1.46	PDGFB	-2.17	MYL7	1.33	PAPSS1
1.45	FGGY			1.33	B3GNT2
1.45	LOC101113041			1.33	TMEM100
1.44	LOC114118747			1.33	ENPP5
1.44	KLF11			1.32	NQO1
1.42	LOC101118514			1.31	CTSB

1.42	IER5			1.29	XIRP2
1.40	TIPARP			1.29	IL27RA
1.38	JUN			1.28	ACTA1
1.38	CCDC197			1.27	UFM1
1.37	IL6R			1.27	ATP6V1A
1.37	C1S			1.27	SNX16
1.37	UBC			1.27	GGT5
1.37	SLC25A33			1.26	MAP3K20
1.36	DYRK3			1.26	PLA2G12A
1.36	PNRC1			1.26	SHC2
1.36	TNNT1			1.25	AZIN1
1.34	UBQLN1			1.25	TEAD4
1.34	SIAH2			1.25	SLC31A1
1.33	OSGIN1			1.25	RHOJ
1.33	LOC114110633			1.23	TRAFD1
1.31	CTSW			1.23	TMED3
1.30	ERF			1.22	CD9
1.29	ARF4			1.22	WFS1
1.29	CCL5			1.21	ITGA7
1.28	BBS2			1.21	SPECC1
1.28	LOC114115357			1.18	NARS
1.27	SHC2			1.17	NPNT
1.27	BHLHE41			1.16	ATP9A
1.27	AFF4			1.16	CTDSP2
1.27	SYNPO2			1.15	STC1
1.26	LIMS1			1.15	SGK1
1.26	NOCT			1.14	ERAP1
1.25	TIMP3			1.14	GJA5
1.25	CDC42EP2			1.14	AQP1
1.24	UCP2			1.14	PDGFC
1.22	TUBA1A			1.14	SORBS2
1.21	SARAF			1.14	SFXN1
1.21	ARPP19			1.13	CAPN2
1.20	MMP2			1.12	YWHAG
1.20	PMP22			1.11	HSPB6
1.20	UBASH3A			1.11	HSPB1
1.20	LYPLA1			1.11	ODF2
1.19	SERPINB1			1.11	ELMOD1
1.19	SLC38A2			1.10	SLC38A2
1.19	LOC106990117			1.10	LOC101102204
1.19	ABHD14B			1.09	SHB

1.19	GRASP			1.09	FLNA
1.18	HMGN2			1.08	SMAD7
1.18	LOC101111528			1.08	FBLN2
1.17	ACTA1			1.08	CXCL12
1.16	PLK2			1.08	WWTR1
1.15	TFF2			1.07	PPP1R14B
1.15	IFITM5			1.07	SYNPO2L
1.13	ACKR3			1.07	SRM
1.13	SOX18			1.05	TPD52
1.12	SQSTM1			1.05	GUCY1B1
1.12	THBS1			1.05	MYLIP
1.12	LOC114117877			1.05	TGFBR2
1.12	IDS			1.04	SPCS3
1.11	ADAMTS1			1.04	CD302
1.11	LOC114111346			1.04	ABTB2
1.11	ZFAND5			1.02	AP1S2
1.11	FSTL3			1.02	STEAP3
1.10	HSF4			1.02	PRELID3B
1.10	CREM			1.02	YIPF5
1.10	GAS1			1.01	KTN1
1.09	KANK1			1.01	KLF2
1.09	MLLT11			1.00	NEK6
1.08	DLL4			1.00	EFHD2
1.07	CRYZ			-1.00	RPS19
1.07	WFS1			-1.00	CKM
1.06	PLP2			-1.00	COQ9
1.06	NEIL2			-1.01	NEMF
1.06	CX3CL1			-1.02	SMDT1
1.06	LOC100101238			-1.03	LOC114109396
1.05	TMEM159			-1.03	THAP7
1.05	LITAF			-1.03	HAND1
1.05	LOC101120481			-1.04	SH3RF2
1.05	CEBPD			-1.04	SESN1
1.05	ENAH			-1.04	TEF
1.05	SLC2A3			-1.04	AKAP1
1.04	NPNT			-1.05	LOC101117028
1.04	XIRP1			-1.05	POLR3H
1.04	CCDC112			-1.05	IVD
1.04	SERINC5			-1.05	LPCAT3
1.04	LOC114108708			-1.06	MYO18B
1.03	GPR157			-1.07	CIB2

1.03	CCDC190			-1.07	ASMTL
1.02	NELFCD			-1.07	GCAT
1.02	NOL3			-1.08	EARS2
1.01	PRSS23			-1.09	FAM81A
1.01	KLHL41			-1.09	DBNDD1
1.00	MAPK1IP1L			-1.09	CRIP2
1.00	SEPTIN9			-1.10	USP2
-1.00	PPP1R3B			-1.11	THAP4
-1.00	THAP4			-1.12	EIF4EBP1
-1.01	LOC114112897			-1.13	GPAT4
-1.02	GRTP1			-1.14	AS3MT
-1.02	MYH7B			-1.14	PABPC4
-1.02	KCNJ5			-1.15	LYVE1
-1.03	LOC114111775			-1.15	IRX5
-1.03	DYNC2LI1			-1.15	LOC101117657
-1.04	THAP3			-1.15	RXRG
-1.06	ANKRD12			-1.15	SLC25A33
-1.06	RBFOX3			-1.16	CSDC2
-1.07	LOC101105958			-1.16	FAM234A
-1.08	CCDC69			-1.16	RPS12
-1.08	MCAT			-1.16	GCSH
-1.08	MBD3			-1.19	LOC114110327
-1.10	PEBP4			-1.19	TRIM63
-1.11	LOC101106131			-1.19	CAVIN4
-1.12	MZT2B			-1.19	ACAP1
-1.12	PDIA2			-1.20	KCNQ1
-1.12	S100A1			-1.20	WBP1
-1.17	LUC7L3			-1.21	GREB1L
-1.17	LOC101123116			-1.22	UROS
-1.18	LOC101112491			-1.22	MCAT
-1.21	LOC101117527			-1.22	CTF1
-1.33	LOC114110252			-1.22	SPR
-1.35	LOC114109394			-1.22	SLC22A3
-1.37	FBXL22			-1.22	FSD2
-1.46	UCHL3			-1.22	ARVCF
-1.50	LOC101120580			-1.23	LOC101114579
-1.51	PKDCC			-1.23	MYL3
-1.54	LOC114113983			-1.24	GYPC
-1.57	ARHGDIG			-1.26	LOC114109394
-1.60	CISH			-1.26	MPND
-1.61	RASL11B			-1.27	FAAP20

-1.61	STARD10			-1.31	PLEKHA6
-1.63	RAD51B			-1.31	LOC114109996
-1.77	ADRB1			-1.32	LOC101106374
-1.78	MAPK12			-1.32	LOC101111664
-1.80	PON3			-1.33	LOC101111733
-2.09	LOC101113217			-1.34	SLC25A34
-2.24	SYN1			-1.35	SLC25A42
-2.70	LOC114108618			-1.36	PHKG1
-2.79	LOC114109396			-1.37	INHA
				-1.38	LENG1
				-1.38	PXMP2
				-1.38	METTL22
				-1.38	SLC38A3
				-1.38	TCEA3
				-1.39	LOC101107260
				-1.41	NUDT8
				-1.42	GTPBP6
				-1.43	PPP1R1A
				-1.44	DUSP8
				-1.44	EGR1
				-1.44	FBXL22
				-1.44	CENPP
				-1.45	LOC101106131
				-1.46	MNS1
				-1.48	MLF1
				-1.48	MYLK3
				-1.48	MDK
				-1.48	UBALD1
				-1.51	PIGZ
				-1.52	MCF2L
				-1.54	DBP
				-1.54	PACRG
				-1.58	LOC101120496
				-1.62	INSYN1
				-1.63	PCDH12
				-1.65	ACSS1
				-1.65	F3
				-1.70	SLC2A4RG
				-1.73	CTH
				-1.76	LOC101105114
				-1.81	HBB

				-1.85	METTL11B
				-1.93	DQA
				-1.94	RAMP1
				-1.95	CHST2
				-1.95	CISH
				-1.95	CITED1
				-2.00	SBK2
				-2.04	FRMD5
				-2.23	LOC101116024
				-2.65	RAD51B
				-2.91	RBFOX3
				-3.00	PDIA2
				-3.04	LOC101107831
				-3.46	LOC101121371

S. Table 2 : Hypertrophy GO analysis by Metascape

	Description	Hits	Log(q-value)
GO Biological Processes	circadian rhythm	ADRB1|CREM|ID1|ID2|ID3|JUN|NFIL3|SIAH2|KLF10|BHLHE40|NOCT|FBXL17|BHLHE41|GPR157|CIART|SIK1|FBXL22	-9.1
GO Biological Processes	blood vessel development	NR4A1|ID1|CCN1|IL6R|JUN|JUNB|SMAD7|MMP2|NPPB|PDGFB|S100A1|SAT1|CX3CL1|THBS1|KLF4|HAND1|ADAMTS1|KLF2|PLK2|TIPARP|SOX18|DLL4|ACKR3|SOX17|APOLD1|NRARP	-7.6
GO Biological Processes	positive regulation of smooth muscle cell proliferation	ID2|IGFBP5|IL6R|JUN|MMP2|PDGFB|CCL5|CX3CL1|THBS1|NR4A3|ADAMTS1	-6.7
GO Biological Processes	muscle structure development	ACTA1|ATF3|CNTFR|FOS|ID2|ID3|IFRD1|IGFBP5|SMAD7|NPPA|PDGFB|MAPK12|TNNT1|BTG2|HAND1|KLHL41|MAFF|DLL4|BHLHE41|ARID5B|SIK1|NPNT	-6.1
Canonical Pathways	PID AP1 PATHWAY	ACTA1|ATF3|DUSP1|FOS|CCN1|JUN|JUNB|MYC|NPPA	-5.9
GO Biological Processes	regulation of ossification	CEBPD|ID1|ID2|ID3|IGFBP5|CCN1|IL6R|SMAD7|SNAI2|NOCT|PKDCC|NPNT|IFITM5	-5.8
GO Biological Processes	response to growth factor	FOS|GAS1|NR4A1|ID1|CCN1|JUN|LIMS1|SMAD7|MYC|PDGFB|CCL5|SNAI2|THBS1|UBC|ZFP36|KLF4|IER2|FSTL3|DDIT4|DLL4|RASL11B|NPNT	-5.6
GO Biological Processes	negative regulation of intracellular signal transduction	ATF3|RCAN1|DUSP1|MYC|NPPA|SNAI2|THBS1|TIMP3|TPT1|WFS1|DYRK3|SOCS1|NOL3|KLF4|LITAF|KANK1|DDIT4|ACKR3	-4.6
GO Biological Processes	regulation of DNA-binding transcription factor activity	FOS|ID1|ID2|ID3|JUN|SMAD7|CX3CL1|SGK1|UBC|WFS1|BHLHE40|KLF4|HAND1|ARID5B|ABRA|SIK1	-4.6
GO Biological Processes	kidney development	ID2|ID3|IL6R|SMAD7|MYC|PDGFB|WFS1|ADAMTS1|FSTL3|TIPARP|SOX17|ARID5B|NPNT	-4.6
GO Biological Processes	fat cell differentiation	ADRB1|BBS2|CEBPD|NR4A1|ID2|SNAI2|ZFP36|NR4A3|SOCS1|KLF4|NOCT|ARID5B	-4.5

GO Biological Processes	epithelial cell proliferation	SERPINB1|NR4A1|ID1|ID2|IGFBP5|JUN|LIMS1|MYC|PDGFB|CCL5|SNAI2|THBS1|ZFP36|NR4A3|DLL4|NRARP	-4.5
GO Biological Processes	positive regulation of cell death	ATF3|NQO1|DUSP1|FOS|ID3|CCN1|JUN|MYC|GADD45B|CCL5|THBS1|TIMP3|UBC|NR4A3|KLF11|SQSTM1|MLLT11|OSGIN1|DDIT4|SIK1	-4.5
GO Biological Processes	extrinsic apoptotic signaling pathway	ATF3|IL6R|LGALS3|CX3CL1|SIAH2|SNAI2|THBS1|TIMP3|NOL3|MLLT11|ITPRIP	-3.8
KEGG Pathway	Fluid shear stress and atherosclerosis	NQO1|DUSP1|FOS|JUN|MMP2|PDGFB|MAPK12|SQSTM1|KLF2	-3.8
GO Biological Processes	Circadian Clock	CREM|NFIL3|UBC|BHLHE40|NOCT|BHLHE41|SIK1	-3.8
GO Biological Processes	negative regulation of cell differentiation	ID1|ID2|ID3|IFRD1|IGFBP5|SMAD7|MYC|PDGFB|PMP22|CX3CL1|SNAI2|ZFP36|SOCS1|FSTL3|PLK2|KANK1|NOCT|BHLHE41|NRARP	-3.7
Canonical Pathways	PID IL6 7 PATHWAY	CEBPD|FOS|IL6R|JUN|JUNB|MYC	-3.6
GO Biological Processes	connective tissue development	BBS2|ID2|CCN1|IL6R|SMAD7|PDGFB|SNAI2|HAND1|ARID5B|PKDCC|MUSTN1	-3.4
GO Biological Processes	heart development	ID1|ID2|ID3|CCN1|JUN|SMAD7|NPPA|PDGFB|SNAI2|HAND1|ADAMTS1|MBD3|SOX18|DLL4|SOX17|SIK1	-3.4

S. Table 3 : Dilated GO analysis by Metascape

Category	Description	Hits	Log(q-value)
GO Biological Processes	cellular response to organic cyclic compound	APLP1|ZFP36L1|COL1A1|ID1|ID3|IGFBP5|TAF7|KLF4|KLF2|DDIT4	-2.9
GO Biological Processes	negative regulation of protein modification process	APOE|CDKN1A|DUSP8|FABP4|LGALS3|RPS3|TAF7|KLF4|PPP1R15A|DDIT4	-2.3
GO Biological Processes	response to toxic substance	APOE|CDKN1A|COL1A1|RPS3|KLF4|KLF2|TXNIP|ADIRF|SRXN1	-2.2
GO Biological Processes	negative regulation of cell differentiation	APOE|ZFP36L1|COL3A1|ID1|ID2|ID3|IGFBP5|NFKBIA|PMP22|S100B	-2.1
GO Biological Processes	response to steroid hormone	ZFP36L1|CDKN1A|COL1A1|S100B|TAF7|TXNIP|DDIT4	-2
Reactome Gene Sets	Scavenging by Class A Receptors	APOE|COL1A1|COL3A1	-2
Reactome Gene Sets	Formation of tubulin folding intermediates by CCT/TriC	CCT6A|TUBA4A|TUBA8	-1.8
GO Biological Processes	intrinsic apoptotic signaling pathway	CDKN1A|RPS3|TPT1|NOL3|PPP1R15A|DDIT4	-1.7
GO Biological Processes	regulation of smooth muscle cell proliferation	CDKN1A|ID2|IGFBP5|NQO2|KLF4	-1.7
GO Biological Processes	muscle structure development	ZFP36L1|COL3A1|ID2|ID3|IGFBP5|S100B|TNNT1|JAM2	-1.5
GO Biological Processes	memory	APOE|MAP1A|NQO2|S100B	-1.3
GO Biological Processes	fat cell differentiation	ZFP36L1|FABP4|ID2|KLF4|ADIRF	-1.3

GO Biological Processes	positive regulation of cell death	CDKN1A|ID3|NQO2|RPS3|S100B|TXNIP|GADD45G|DDIT4	-1.3
GO Biological Processes	cold-induced thermogenesis	FABP4|ID1|SLN|GADD45G	-1.2
GO Biological Processes	protein localization to nucleus	CCT6A|CDKN1A|COL1A1|NFKBIA|TXNIP	-1.2
GO Biological Processes	negative regulation of cell adhesion	ARHGDIG|COL1A1|LGALS3|KLF4|JAM2	-1.1
GO Biological Processes	supramolecular fiber organization	APOE|COL1A1|COL3A1|ID1|MAP1A|RPS3|TNNT1	-0.9
GO Biological Processes	regulation of cellular amide metabolic process	APOE|ZFP36L1|IGFBP5|RPS3|PPP1R15A|NGRN	-0.88
GO Biological Processes	PI3K-Akt signaling pathway	CDKN1A|COL1A1|VWF|DDIT4|THEM4	-0.86
Reactome Gene Sets	Transcriptional regulation of white adipocyte differentiation	FABP4|KLF4|ADIRF	-0.85

S. Table 4 : Failure GO analysis by metascape

Category	Description	Hits	Log(q-value)
GO Biological Processes	muscle structure development	ACTA1|ATF3|ZFP36L1|CAPN2|CD9|CTF1|CTH|EGR1|ITGA7|SMAD7|MYL3|SORT1|S100B|TEAD4|TGFBR2|TNNT1|SORBS2|USP2|HAND1|ANKRD2|PDLIM3|SYNPO2L|MYO18B|MYLK3|XIRP2|NPNT|CAVIN4	-6.3
GO Biological Processes	blood vessel morphogenesis	AQP1|ZFP36L1|F3|GJA5|HIF1A|HSPB1|ITGAV|LRP1|SMAD1|SMAD7|MDK|MMP2|CITED1|SHB|STAT3|TGFBR2|THBS1|HAND1|ADAMTS1|RAMP1|KLF2|ERAP1|TMEM100|RHOJ|MYO18B|HSPB6	-5.5
GO Biological Processes	apoptotic signaling pathway	ATF3|CD44|CTH|CYLD|HIF1A|HSPA1A|HSPB1|ITGAV|LGALS3|SRGN|SORT1|CXCL12|SNAI2|THBS1|TIMP3|TNFRSF1B|TPT1|WFS1|YWHAG|BAG3|ANKRD2|UBQLN1	-4.4
GO Biological Processes	response to wounding	ACTN1|ARF4|ZFP36L1|CD9|CD44|F3|FLNA|HBB|HIF1A|HSPB1|LRP1|MDK|SERPINE2|RAD51B|TGFBR2|THBS1|TIMP1|VWF|AKAP1|PABPC4|LYVE1|MMRN1|SULF2	-4
GO Biological Processes	regulation of muscle system process	CNN1|FLNA|GJA5|KCNQ1|SMAD7|MYL3|SLN|STC1|TNFRSF1B|TNNT1|ERRFI1|TRIM63|HSPB6|NPNT	-4
GO Biological Processes	cellular response to organonitrogen compound	ACTA1|AQP1|ATP6V1A|ZFP36L1|CAPN2|EGR1|EIF4EBP1|KCNQ1|LRP1|MMP2|ENPP1|STAT3|STC1|UROS|YWHAG|KLF2|CIB2|SESN1|ERRFI1|PDGFC|SLC25A33	-4
GO Biological Processes	response to oxygen levels	AQP1|ATP6V1A|ZFP36L1|CAPN2|EGR1|EIF4EBP1|HIF1A|LDHA|MMP2|S100B|CXCL12|STC1|TGFBR2|THBS1|UCP2|UBQLN1	-3.5
GO Biological Processes	response to oxidative stress	AQP1|CAPN2|NQO1|GUCY1B1|HBB|HIF1A|HSPA1A|HSPB1|LDHA|MMP2|PON2|KLF2|ANKRD2|SESN1|UBQLN1|STK26|SRXN1	-3.4
GO Biological Processes	skeletal system development	CD44|GJA5|HIF1A|INHA|SMAD1|SMAD7|MDK|MMP2|SNAI2|STC1|TEAD4|TGFBR2|TIMP1|PAPSS1|HAND1|IRX5|SULF2|PDGFC	-3.4
GO Biological Processes	negative regulation of phosphate metabolic process	ATF3|CYLD|DUSP8|FABP4|HSPB1|INHA|LGALS3|SMAD7|ENPP1|PPP1R1A|STAT3|TIMP3|YWHAG|CTDSP2|WWTR1|PPP1R14B|ERRFI1|DBNDD1|SH3RF2	-3.2

Reactome Gene Sets	extracellular matrix organization	CAPN2|CD44|FBLN2|ITGA7|ITGAV|LRP1|MMP2|THBS1|TIMP1|TNFRSF1B|VWF|ADAMTS1|SULF2|SLC39A8|NPNT	-3
GO Biological Processes	actin cytoskeleton organization	ACTA1|ACTN1|AQP1|CNN1|FLNA|LRP1|MDK|CXCL12|TNNT1|SORBS2|PDLIM3|ENAH|ACTR3B|RHOJ|SYNPO2L|FRMD5|MYLK3|SPECC1|XIRP2|SYNPO2	-3
GO Biological Processes	Ferroptosis	F3|ACSL4|LPCAT3|SLC38A2|STEAP3|SLC39A8	-3
CORUM	VEGF transcriptional complex	HIF1A|CITED1|STAT3	-2.9
GO Biological Processes	extrinsic apoptotic signaling pathway	ATF3|CYLD|HSPA1A|ITGAV|LGALS3|SORT1|SNAI2|THBS1|TIMP3|TNFRSF1B|BAG3	-2.8
GO Biological Processes	transmembrane receptor protein tyrosine kinase signaling pathway	ARF4|ATP6V1A|EIF4EBP1|F3|HIF1A|HSPB1|IGFBP4|ITGAV|LRP1|MMP2|ENPP1|SORT1|SHB|STAT3|THBS1|SLC9A6|SHC2|ERRFI1|SULF2|PDGFC	-2.6
GO Biological Processes	reactive oxygen species metabolic process	ARF4|NQO1|HBB|HIF1A|SPR|STAT3|TGFBR2|THBS1|STK17A|KLF2|SESN1|SLC25A33	-2.6
GO Biological Processes	cellular response to growth factor stimulus	ZFP36L1|CD44|EGR1|HIF1A|HSPA1A|HSPB1|SMAD1|SMAD7|CITED1|SORT1|SNAI2|TGFBR2|THBS1|TNFRSF1B|SLC9A6|ERRFI1|TMEM100|SULF2|NPNT	-2.5
GO Biological Processes	renal system process	AQP1|GJA5|HBB|KCNQ1|SGK1|STC1|WFS1|SULF2	-2.4
GO Biological Processes	anion transport	AQP1|FABP4|ACSL4|HBB|ENPP1|SLC1A5|SLC22A3|STC1|THBS1|ATP9A|SLC9A6|SLC38A3|PRELID3B|SLC38A2|SLC39A8|PLA2G12A|SFXN1|SLC25A42	-2.4

S. Table 5 : Diseases associated with significantly expressed genes in Hypertrophy stage

Description	Hits	Log(q-value)
Endotoxemia	ATF3|CREM|SERPINB1|FOS|NR4A1|IGFBP5|IL6R|JUN|JUNB|LGALS3|MMP2|CCL5|CX3CL1|TIMP3|SQSTM1|ADAMTS1|KLF2|DDIT4|ACKR3|SOX17	-8.7
Anoxia	DUSP1|FOS|CCN1|JUN|MYC|NPPA|PDGFB|CX3CL1|SGK1|SIAH2|SLC2A3|THBS1|UCP2|BHLHE40|DDIT4|PDIA2|BHLHE41	-8.2
Myocardial Ischemia	ADRB1|CEBPD|RCAN1|DUSP1|NR4A1|ID1|ID3|IFRD1|CCN1|JUNB|LGALS3|NPPA|NPPB|RAD51B|CX3CL1|KLF10|UCP2|ZFP36|TUBA1A|BHLHE40|KLF4|PRSS23|DDIT4|DLL4	-7
Vascular inflammations	DUSP1|FOS|NR4A1|JUN|JUNB|LGALS3|SMAD7|MMP2|CX3CL1|SGK1|TIMP3|ZFP36|KLF4|KLF2|DDIT4|DLL4	-6.9
Cardiomyopathy, Familial Idiopathic	ADRB1|DUSP1|FOS|KCNJ5|LGALS3|MMP2|MYC|NPPA|NPPB|S100A1|SNAI2|THBS1|TIMP3|UCP2|SQSTM1|KLF4|HAND1|SOX18|ACKR3|CSRNP1|ABRA|SIK1|XIRP1	-6.1
Polyarthritis, Juvenile, Rheumatoid Factor Negative	CREM|DUSP1|FOS|JUN|SLC2A3|THBS1|NR4A3|KLF4|PLK2|MAFF|SIK1	-6.1
Polyarthritis, Juvenile, Rheumatoid Factor Positive	CREM|DUSP1|FOS|JUN|SLC2A3|THBS1|NR4A3|KLF4|PLK2|MAFF|SIK1	-6.1
Juvenile-Onset Still Disease	CREM|DUSP1|FOS|IL6R|JUN|SLC2A3|THBS1|NR4A3|KLF4|PLK2|MAFF|SIK1	-6
Juvenile psoriatic arthritis	CREM|DUSP1|FOS|JUN|SLC2A3|THBS1|NR4A3|KLF4|PLK2|MAFF|SIK1	-5.9
Lung diseases	NQO1|DUSP1|SERPINB1|FOS|NR4A1|IFRD1|IGFBP5|JUN|JUNB|LGALS3|SMAD7|MMP2|CCL5|CX3CL1|TFF2|KLF10|TIMP3|KLF2|AFF4|ACKR3|NPNT	-5.5
Diabetes Mellitus, Experimental	ADRB1|ATF3|NQO1|DUSP1|ID1|LGALS3|MMP2|MYC|NPPA|NPPB|PDGFB|CX3CL1|SLC2A3|THBS1|UCP2|IRS2|LITAF|SLC38A2	-5.3
Reperfusion Injury	ADRB1|ATF3|FOS|ID3|IL6R|JUN|SAT1|CCL5|CX3CL1|SLC2A3|THBS1|TIMP3|BTG2|SOCS1	-5.2
Vascular Diseases	FOS|NR4A1|IGFBP5|CCN1|IL6R|JUN|JUNB|LGALS3|MMP2|NPPB|S100A1|SGK1|THBS1|UCP2|KLF4|KLF2|SOX18|DLL4|ACKR3|SYNPO2	-5

Pancreatic Neoplasm	NQO1|DUSP1|GEM|NR4A1|ID1|CCN1|IL6R|JUN|LGALS3|SMAD7|MMP2|MYC|S100A1|TFF2|THBS1|BHLHE40|SOCS1|IRS2|KLF4|LYPLA1|MCAT	-5
Juvenile arthritis	CREM|DUSP1|FOS|GEM|IL6R|JUN|MMP2|S100A1|CCL5|SLC2A3|THBS1|NR4A3|KLF4|PLK2|MAFF|SIK1	-4.7
Immune System Diseases	CISH|RCAN1|FOS|GEM|NR4A1|JUN|JUNB|PMP22|S100A1|CCL5|CX3CL1|THBS1|NR4A3|SOCS1|KLF4|UBASH3A	-4.7
Classical Hodgkin's Lymphoma	ATF3|CEBPD|FOS|GEM|ID2|JUN|JUNB|MYC|CCL5|TIMP3|SOCS1|KLF4|SEPTIN9	-4.6
Helicobacter pylori (H. pylori) infection in conditions classified elsewhere and of unspecified site	ATF3|CRYZ|NQO1|FOS|IDS|JUN|JUNB|LGALS3|SMAD7|MYC|CCL5|SGK1|SIAH2|TFF2|THBS1|SQSTM1|NOL3|KLF4	-4.6
Impaired glucose tolerance	ATF3|CISH|SERPINB1|IGFBP5|LGALS3|SMAD7|NFIL3|SAT1|CCL5|SGK1|SLC2A3|THBS1|KLF10|UCP2|WFS1|NR4A3|KLF11|IRS2	-4.6
Respiratory syncytial virus (RSV) infection in conditions classified elsewhere and of unspecified site	CISH|CRYZ|NQO1|DUSP1|FOS|JUN|MMP2|CCL5|CX3CL1|THBS1|ZFP36|SOCS1|DLL4|PDIA2|SLC25A33|RBFOX3	-4.5

S. Table 6 : Diseases associated with significantly expressed genes in Dilated stage

Description	Hits	Log(q-value)
Myocardial Ischemia	APOE|FABP4|GPT|ID1|ID3|LGALS3|NFKBIA|SLN|VWF|KLF4|TXNIP|PPP1R15A|DDIT4|SRXN1	-5.3
Comedone	CISH|LGALS3|S100B|VWF|TXNIP	-3.3
Stable angina	APOE|COL3A1|FABP4|LGALS3|VWF|KLF2	-2.9
Noninfiltrating Intraductal Carcinoma	CDKN1A|CISH|COL1A1|GPT|LGALS3|S100B|SLN|VWF|TXNIP	-2.9
Hypercholesterolemia	APOE|COL3A1|GPT|ID2|LGALS3|VWF|SUCLA2|KLF2|DDIT4	-2.9
Sleep Apnea Syndromes	APOE|COL3A1|LGALS3|PMP22|S100B|VWF	-2.9
Ureteral obstruction	COL1A1|COL3A1|ID1|LGALS3|S100B|KLF4|TXNIP|GADD45G	-2.9
Subarachnoid Hemorrhage	APOE|COL3A1|GPT|LGALS3|S100B|VWF|KLF4|TXNIP|DDIT4	-2.9
Diabetes Mellitus, Experimental	APOE|CDKN1A|FABP4|ID1|LGALS3|NFKBIA|S100B|VWF|TXNIP	-2.8
Nonalcoholic Steatohepatitis	APOE|CDKN1A|COL1A1|FABP4|GPT|LGALS3|KLF4|TXNIP	-2.4
Decompensated cirrhosis of liver	FABP4|GPT|LGALS3|VWF	-2.2
Intervertebral Disc Degeneration	APOE|COL1A1|COL3A1|IGFBP5|LGALS3|TXNIP|DDIT4	-2.1
Marfan Syndrome	COL1A1|COL3A1|SLN|VWF|KLF4	-2.1
Pneumonitis	APOE|COL1A1|COL3A1|FABP4|GPT|LGALS3|S100B|VWF|KLF4	-1.9
Drug-Induced Liver Disease	APOE|COL3A1|FABP4|GPT|LGALS3|S100B|VWF|SRXN1	-1.9
Progressive Neoplastic Disease	APOE|GPT|NFKBIA|PMP22|S100B|VWF|KLF2	-1.9
Progressive cGVHD	APOE|GPT|NFKBIA|PMP22|S100B|VWF|KLF2	-1.9
Anaplastic thyroid carcinoma	APOE|CDKN1A|COL1A1|LGALS3|KLF4|TXNIP|GADD45G	-1.9
Complete atrioventricular block	CISH|FABP4|GPT|HSF4|LGALS3|VWF|NOL3	-1.8
Pulmonary Hypertension	APOE|CDKN1A|COL1A1|ID1|VWF	-1.8

S. Table 7 : Diseases associated with significantly expressed genes in Failure stage

Description	Hits	Log(q-value)
Cardiomyopathy, Familial Idiopathic	CAPN2|CNN1|CTH|CTSB|F3|FABP4|HIF1A|LGALS3|MMP2|MYL3|PPP1R1A|SRGN|S100B|CXCL12|SLC22A3|SNAI2|STAT3|THBS1|TIMP1|TIMP3|TNFRSF1B|UCP2|HAND1|BAG3|MMRN1|PDLIM3|SULF2|RHOJ|MYLK3|SFXN1|HSPB6|XIRP2|ABRA	-9.4
Vascular Diseases	CAPN2|CTH|EGR1|F3|FLNA|HIF1A|HSPA1A|HSPB1|LGALS3|MMP2|SORT1|S100B|CXCL12|SGK1|STAT3|TGFBR2|THBS1|TIMP1|TNFRSF1B|UCP2|VWF|LPCAT3|KLF2|MMRN1|MYLIP|PDGFC|TRIM63|SYNPO2	-7.1
Middle Cerebral Artery Occlusion	AQP1|ATF3|CTSB|CYLD|EGR1|HIF1A|HSPA1A|HSPB1|LDHA|LGALS3|LRP1|MDK|MMP2|S100B|CXCL12|SGK1|STAT3|TIMP1|UCP2|VWF|AKAP1|ADAMTS1|KLF2|UBQLN1|STK26|SRXN1	-6.8
Complications of Diabetes Mellitus	ATF3|CYLD|EGR1|HIF1A|HSPB1|KCNQ1|LGALS3|SMAD1|MMP2|ENPP1|PON2|CXCL12|STAT3|THBS1|TPD52|UCP2|MMRN1	-6.8
Aortic Aneurysm, Abdominal	AQP1|CD44|CTSB|EGR1|F3|FABP4|FBLN2|FLNA|HIF1A|LGALS3|LRP1|MDK|MMP2|SORT1|CXCL12|STAT3|TGFBR2|THBS1|TIMP1|TIMP3|UCP2|VWF|ADAMTS1|MMRN1|MCAT	-6.8
Left Ventricular Hypertrophy	ACTA1|CKM|CTF1|DBP|NQO1|FABP4|GJA5|HIF1A|LGALS3|MMP2|MYL3|ENPP1|PON2|S100B|CXCL12|THBS1|TIMP1|AKAP1|ERAP1|COQ9	-6
Cardiomyopathy, Dilated	ACTA1|CNN1|CTSB|F3|GJA5|ITGA7|LDHA|LGALS3|CXCL12|STAT3|TIMP1|TIMP3|TNFRSF1B|HAND1|BAG3|MMRN1|PDLIM3|UFM1|MAP3K20|HSPB6|ABRA|CAVIN4	-5.8
Heart Diseases	CTF1|NQO1|F3|GJA5|HIF1A|KCNQ1|LGALS3|MMP2|MYL3|PON2|CXCL12|SRM|STAT3|THBS1|TIMP1|TIMP3|VWF|HAND1|ADAMTS1|MMRN1|UBQLN1|HSPB6	-5.5
Marfan Syndrome	CD44|CNN1|KCNQ1|LRP1|MMP2|SLN|TGFBR2|THBS1|TIMP3|VWF|ADAMTS1|MMRN1	-5.5
Secondary malignant neoplasm of bone	ATF3|CD44|CTSB|EGR1|F3|FABP4|HIF1A|HSPB1|ITGAV|SMAD7|MMP2|ENPP1|CXCL12|SGK1|SNAI2|STAT3|ADAMTS1|RAMP1|MMRN1|WWTR1|PDLIM3|MCAT|MAP3K20|NPNT	-5.5
Reperfusion Injury	ATF3|CD44|CTSB|EGR1|HIF1A|HSPA1A|HSPB1|SERPINE2|S100B|CXCL12|STAT3|STC1|TGFBR2|THBS1|TIMP3|BAG3	-4.9
Lung diseases	CD9|CD44|CTH|CTSB|CYLD|NQO1|EGR1|FLNA|HIF1A|IGFBP4|KCNQ1|LGALS3|SMAD7|MMP2|CXCL12|STAT3|STC1|TIMP1|TIMP3|AP1S2|IRX5|KLF2|MMRN1|NPNT	-4.9

Acute Coronary Syndrome	CKM|CTSB|DBP|EGR1|F3|FABP4|HSPA1A|IGFBP4|LGALS3|LRP1|MMP2|CXCL12|STAT3|TIMP1|UCP2|VWF|KLF2|MMRN1|FRMD5	-4.9
Pancreatic Neoplasm	CD44|CTSB|NQO1|F3|HIF1A|LDHA|LGALS3|SMAD1|SMAD7|MDK|MMP2|SERPINE2|S100B|CXCL12|STAT3|TGFBR2|THBS1|TIMP1|SORBS2|BAG3|B3GNT2|LYVE1|MMRN1|WWTR1|MCAT	-4.9
Ischemia	CKM|EGR1|HIF1A|ITGAV|MMP2|PON2|S100B|CXCL12|STAT3|THBS1	-4.5
Myopathy	ACTA1|ACTN1|CAPN2|CTSB|FLNA|HIF1A|HSPB1|ITGA7|LDHA|MMP2|RPS19|SLN|STAT3|THBS1|TNFRSF1B|TNNT1|WFS1|AP1S2|BAG3|TRIM63|MYO18B|CAVIN4	-4.4
Acute pancreatitis	CTH|CTSB|CYLD|NQO1|EGR1|F3|HIF1A|HSPA1A|HSPB1|LGALS3|SMAD7|MDK|MMP2|CXCL12|STAT3|UCP2|RAMP1|KLF2	-4.3
Atherosclerotic lesion	AQP1|CTH|EGR1|FABP4|HSPB1|LGALS3|MDK|MMP2|SORT1|CXCL12|TGFBR2|UCP2|KLF2|MMRN1	-4.3
Hypercholesterolemia	ATF3|CTF1|F3|ACSL4|FLNA|HIF1A|HSPA1A|LGALS3|PON2|SORT1|CXCL12|STAT3|THBS1|TNFRSF1B|VWF|KLF2|MMRN1|MYLIP|ENAH	-4.3
Transient Ischemic Attack	CTSB|NQO1|F3|HIF1A|HSPA1A|HSPB1|MMP2|CXCL12|SGK1|STAT3|TGFBR2|THBS1|TNFRSF1B|UCP2|VWF|ADAMTS1	-4.3

S. Table 8. Potential cardiovascular disease genes expressed in the cardiac hypertrophy stage

log2 Fold Change(Hypertrophy/Control)	Genes in HYPERTROPHY previously not reported in CVD	Description
3.38	OTUD1	OTU deubiquitinase 1;
2.28	C15H11orf96	chromosome 15 C11orf96 homolog
2.09	OVAR-DRB1	DLA class II histocompatibility antigen, DR-1 beta chain-like
2.09	ARID5A	AT-rich interaction domain 5A
1.98	CIART	circadian associated repressor of transcription
1.87	NRARP	NOTCH regulated ankyrin repeat protein
1.67	MUSTN1	musculoskeletal, embryonic nuclear protein 1
1.67	DZANK1	double zinc ribbon and ankyrin repeat domains 1
1.66	FBXL17	F-box and leucine rich repeat protein 17
1.63	NARS	Asparaginyl-tRNA synthetase 1
1.52	OVAR	MHC class I antigen
-1.51	PKDCC	protein kinase domain containing, cytoplasmic
-1.57	ARHGDIG	Rho GDP dissociation inhibitor gamma
-1.61	STARD10	StAR related lipid transfer domain containing 10
-1.78	MAPK12	mitogen-activated protein kinase 12

S. Table 9. Potential cardiovascular disease genes expressed in the myocardium dilatation stage

log2 Fold Change(Dilated/Control)	Genes in DILATED stage previously not reported in CVD	Description
2.05	TMEM205	transmembrane protein 205
1.99	ADIRF	adipogenesis regulatory factor
1.84	C6H4orf48	chromosome 6 C4orf48 homolog
1.74	NGRN	neugrin, neurite outgrowth associated
1.71	PROSER1	proline and serine rich 1
1.68	ERICH2	glutamate rich 2
1.54	C15H11orf96	chromosome 15 C11orf96 homolog
-1.65	ARHGDIG	Rho GDP dissociation inhibitor gamma

S. Table 10. Potential cardiovascular disease genes expressed in the heart failure stage

log2 Fold Change(Failure/Control)	Genes in FAILURE stage previously not reported in CVD	Description
2.91	ANKRD2	ankyrin repeat domain 2
2.22	STK38L	serine/threonine kinase 38 like
1.98	ACOT9	acyl-CoA thioesterase 9
1.80	ARID5A	AT-rich interaction domain 5A
1.73	FKBP10	FKBP prolyl isomerase 10
1.63	OLFML3	olfactomedin like 3
1.57	LITAF	lipopolysaccharide induced TNF factor
1.52	SKP1-2	SKP1 S-phase kinase associated protein 1
-1.51	PIGZ	phosphatidylinositol glycan anchor biosynthesis class Z
-1.54	PACRG	parkin coregulated
-1.62	INSYN1	inhibitory synaptic factor 1
-1.85	METTL11B	methyltransferase like 11B
-1.93	DQA	SLA class II histocompatibility antigen, DQ haplotype D alpha chain
-1.95	CHST2	carbohydrate sulfotransferase 2
-1.95	CITED1	Cbp/p300 interacting transactivator with Glu/Asp rich carboxy-terminal domain 1
-2.00	SBK2	SH3 domain binding kinase family member 2
-2.91	RBFOX3	RNA binding fox-1 homolog 3
