# Supplementary figures and images for: Transcriptomal Insights of Heart Failure from Normality to Recovery
Source: Biomolecules. 2022 May 23;12(5):731. doi: 10.3390/biom12050731 (PMC9138767; doi:10.3390/biom12050731)

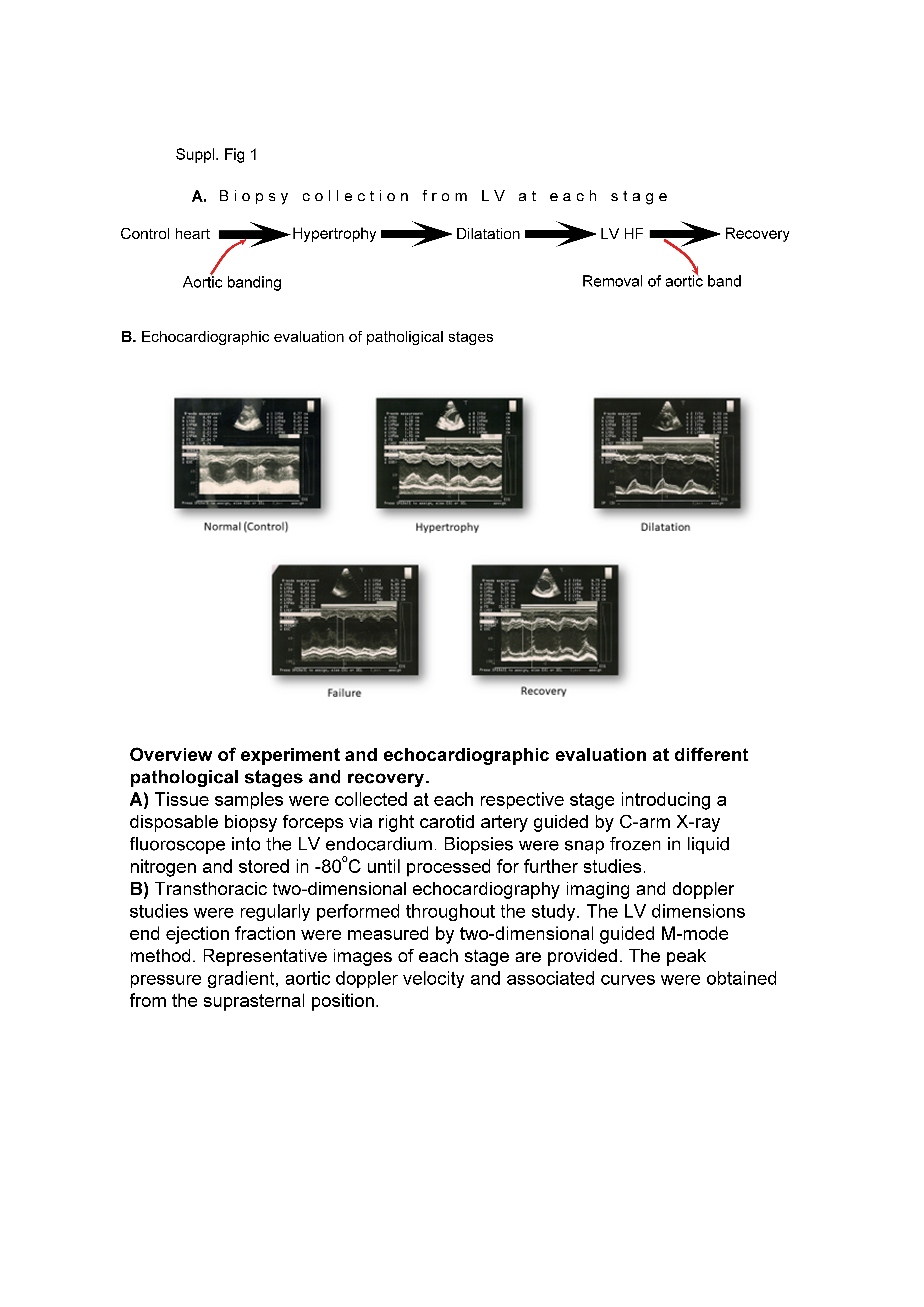

Supplement: Supplementary file 1 [file biomolecules-12-00731-s001.zip › Supplementary Figure S1.jpg]
